# Enhancement of Fear Extinction Memory and Resistance to Age-Related Cognitive Decline in Butyrylcholinesterase Knockout Mice and (*R*)-Bambuterol Treated Mice

**DOI:** 10.3390/biology10050404

**Published:** 2021-05-05

**Authors:** Weiwei Liu, Yan Cao, Yue Lin, Keai Sinn Tan, Haishan Zhao, Haihua Guo, Wen Tan

**Affiliations:** 1 Institute of Biomedical and Pharmaceutical Sciences, Guangdong University of Technology, Guangzhou 510006, China; aireou@mail2.gdut.edu.cn (W.L.); catbrighton@163.com (Y.L.); hartzhao@163.com (H.Z.); Guohaihua2021@163.com (H.G.); 2 YZ Health-tech Inc., Hengqin District, Zhuhai 519000, China; caoyanjoy@163.com (Y.C.); tankeaisinn@jnu.edu.cn (K.S.T.); 3 School of Pharmacy, Jinan University, Guangzhou 510632, China; 4 Jeffrey Cheah School of Medicine and Health Sciences, Monash University Malaysia, Bandar Sunway 47500, Malaysia

**Keywords:** butyrylcholinesterase, (*R*)-bambuterol, fear extinction memory, cognitive decline

## Abstract

**Simple Summary:**

Fear extinction is the driving mechanism to reduce the fear response, and it is the basis of exposure-based cognitive-behavioral therapy. Butyrylcholinesterase (BChE) seems to be involved in regulating cognitive function, and its relationship with fear extinction memory has not been reported. BChE knockout mice and wild-type mice with administration of (*R*)-bambuterol, a BChE inhibitor, were used in this study. In addition to immunohistochemistry and metabolite analysis using mass spectrometry imaging, the influence of age on the conditioned fear test, Morris water maze experiment, and open field test were carefully evaluated. Our results showed that BChE inhibition accelerates the fear extinction memory in mice and delays the cognitive decline in the early stages of aging.

**Abstract:**

Butyrylcholinesterase (BChE) is detected in plaques preferentially in Alzheimer’s disease (AD) and may be associated with stress disorders. However, the physiological function of BChE in the central nervous system remains to be further investigated. BChE knockout (KO) mice and wild-type (WT) mice with orally or intranasal administration of (*R*)-bambuterol were used to explore the effect of BChE on behavior changes. (*R*)-bambuterol is a specific and reversible inhibitor of BChE. The behavior changes were evaluated and compared among 3–10 month old mice. Our finding showed that BChE KO and (*R*)-bambuterol administration enhanced episodic memory, including fear conditioning memory and fear extinction memory in fear conditioning and fear extinction test. BChE KO and (*R*)-bambuterol administered mice rescued age-related spatial memory and general activity in the water maze test and open field test. The brain metabolomics were imaged using a desorption electrospray ionization mass spectrometry imaging (DESI-MSI). The image of DESI-MS demonstrated that glutamine content increased in the brain of BChE KO mice. In conclusion, this study found that inhibition of BChE ameliorated episodic and spatial memories. This study also suggested that (*R*)-bambuterol as a BChE inhibitor has the potential application in the treatment of post-traumatic stress disorder (PTSD) and early cognitive decline.

## 1. Introduction

Butyrylcholinesterase (BChE; EC 3.1.1.8), a nonspecific cholinesterase (ChE) or pseudocholinesterase involved in breaking down esters of choline, is widely distributed in the central nervous system and peripheral tissues in humans [1,2]. BChE was once considered to have no physiological function, given that people with loss of the enzymatic activity of BChE appear to be completely normal [3]. BChE took part in the various physiological process [4,5]. The sensitivity of BChE to organophosphorus poisons, therapeutic effects of BChE on drug addicts [4,6], and the susceptibility to high-fat diet-induced obesity in BChE knockout (KO) mice [7,8] were reported. A decrease in BChE levels results in an increased sensitivity to anesthetic agents (e.g., succinylcholine and mivacurium) [9]. 

Acetylcholine (Ach), a substrate of ChE, exhibits a vital role in cognitive functions’ neuromodulation [10]. ChE inhibitors (e.g., selective AChE inhibitors donepezil, galantamine, and nonselective ChEs inhibitor rivastigmine) were used to enhance the pathologically reduced ACh levels. These drugs were approved by the Food and Drug Administration (FDA) to treat neurodegenerative disease such as Alzheimer’s disease (AD). However, these drugs have shown modest cognitive benefits in demented patients, with no consistent therapeutic outcomes and develop side effects [11]. As such, the clinical significance of these drugs is unclear [12]. Therefore, using a selective inhibition of BChE to treat neurodegenerative diseases should be the focus [7,10].

Aging is a significant risk factor for most neurodegenerative diseases, which affects the quality of life of the elderly [13]. The enzymatic activity of BChE progressively increases in AD patients, which is closely associated with aging and the decomposition of β-amyloid (Aβ) plaques [14,15]. As a biomarker, BChE can predict the progression of neurodegenerative diseases [16]. Therefore, we speculate that BChE is closely associated with aging. An increase in ghrelin level can eliminate fear and inhibit the recurrence of fear [17], and BChE can hydrolyze ghrelin [18]. Failure to eliminate fear is a hallmark of stress disorders and neuropsychiatric diseases [19]. BChE is involved in the metabolism of endocannabinoids [20], and endocannabinoids are negatively correlated with the development of post-traumatic stress disorder (PTSD) [21]. Following that, we hypothesize that BChE inhibition may manifest a therapeutic role in age-related cognitive decline as well as in stress disorders such as PTSD [22].

Bambuterol is a potent pseudo-substrate inhibitor of BChE with a half-maximal inhibitory concentration (IC_50_) of 3 × 10^−9^ M. It is also a weak AChE inhibitor with IC_50_ = 3 × 10^−5^ M. The BChE inhibition rate constant of bambuterol is about 18,000 times higher than that of rivastigmine [23]. The enzymatic activity of BChE is inhibited by bambuterol enantiomers, with a preference for the (*R*)-bambuterol in which the inhibition rate constants are approximately 5-times higher than (*S*)-bambuterol [24]. Because of that, (*R*)-bambuterol is a suitable drug candidate to inhibit BChE activity. However, it is difficult for bambuterol to penetrate the blood-brain barrier. A recent study reports that intranasal administration of bambuterol can enter both the circulatory system and the central nervous system [23]. 

To explore the effect of BChE on age-related cognitive decline and PTSD, BChE KO and WT mice with oral or intranasal administration of (*R*)-bambuterol were studied. Our study suggested that (*R*)-bambuterol, as a selective BChE inhibitor, possibly benefit neurodegeneration associated with aging and stress disorders.

## 2. Methods

### 2.1. Mouse Genotyping

Two-month-old wild-type (WT) C57BL/6 female mice were purchased from Guangzhou University of Chinese Medicine. BChE knockout (KO) male mice used for breeding were constructed in our laboratory [25]. All mice were kept in an SPF-grade animal room at 25 °C on a 12-h light-dark cycle, free access to standard pellet diet and water. WT female mice and BChE KO male mice were combined and mated in the same cage at the ratio of 2:1. Male mice were separated three days later, and the pregnant female mice were reared in separate cages two weeks later. Then the obtained heterozygous KO mice of the F1 generation were selected to mate with each other to generate F2 generation mice of various genotypes, including wild type (WT), heterozygous KO (BChE^+/−^), and homozygous KO (BChE^−/−^), which used in this study. The genotypes of F2 generation mice were identified by gene amplification and sequencing. The BChE sequence of the experimental mouse was NM_009738.3 in GenBank, and the KO site was 5′-CCCTATGCACAACCTCCTC-3′. The primer synthesis and amplification sequencing were provided by Shenggong Bioengineering (Shanghai, China) Co., Ltd. The primers were as Table 1:

### 2.2. Intranasal and Oral Administration of (R)-Bambuterol in WT C57BL/6 Mice

(*R*)-bambuterol (R-BMB, purity ≥ 99.0%) used in this study was synthesized in our laboratory. (*R*)-bambuterol (3 mg/kg, once a day) was given to male C57BL/6 mice intranasally (WT-ND group) or orally (WT-OA group) for 7 days, and (*R*)-bambuterol was administered throughout the experiments. Due to the small volume of nasal drops, the concentration of (*R*)-bambuterol in saline was 7.5 mg/mL for the WT-ND group and 0.3 mg/mL for WT-OA group.

### 2.3. Animal Experiment Design and Age Description

Three-month-old and four-month-old mice are sexually matured and were used as young mice in this study. While 10-month-old mice were chosen as mice in early old age, because they exhibited a mild cognitive decline in our study. The age of mice increased during the experiments including fear conditioning and fear extinction test, Morris water maze test and open field test. Therefore, mice at the age of 6-month, 7-month, and 8-month were used in our study. Table 2 summarized the ages of mice used in each experiment. 

### 2.4. BChE Enzymatic Activity Determination

Serum BChE activity was detected using Butyrylcholinesterase assay kit (Nanjing Jiancheng Bioengineering Institute, Nanjing, China), following the manufacturer’s instructions. The enzymatic reaction rate of BChE was measured using the Ellman method [26]. In a 96-well plate, 10 μL of serum was added into phosphate buffer (215 μL, pH 8.0, 100 mmol/L) and incubated at 37 °C for 5 min. Following that, 25 μL of 37 °C preheated MIX Buffer (5,5′-dithiobis-2-nitrobenzoic acid, DTNB, 3.4 mmol/L; butyrylthiocholine iodide, BTCh, 2.5 mmol/L) was added and mixed well. The mixed solution was immediately placed in a 37 °C microplate reader (Berthold Technologies, TriStar2S LB942, Bad Wildbad, Germany) and measured at 412 nm every 2–3 min (starting at 5 min and ending at 30 min). 

### 2.5. Fear Conditioning and Fear Extinction Test

The fear conditioning and extinction experimental parameters were set according to previous publications with modifications [27,28,29]. In our experiments, the conditioned stimulus (CS) were sound (80 dB intensity, 360 Hz frequency, for 40 s) and light (80 Cd intensity, 505 Hz optical frequency, for 27 s); the followed unconditioned stimulus (US) was scrambled foot-shock treatment (0.5 mA intensity, for 3 s). Freezing was defined as complete immobility of the body and head with no interaction with the environment. Fear memory was evaluated by measuring freezing time [29]. 

Procedure for fear conditioning training (CS + US): each male mouse was placed into a box with an environmental adaptation period of 180 s before the experiment; then the sound stimulus started at 0 s; the light stimulus started at 10s; the electric foot shock started at 37 s; the experiment cycle was 60 s; 2 continuous cycles was performed once a day. The mice were stimulated with CS + US for 3 consecutive days.

Procedure for re-exposure to fear conditioning training: the mice were stimulated with CS + US on day 1–3, and after 72 hours, they were stimulated with CS + US on day 6–7. 

Procedure for fear extinction training: after the mice were stimulated with CS + US on day 1–2, they were stimulated with CS for 3 or 4 consecutive days.

Procedure for combination of fear conditioning, re-exposure and extinction training: The mice were stimulated with CS + US on days 1–3, and after the test was stopped for 6 days, they were stimulated with CS + US on days 9–10 and CS stimulation on days 11–14. 

The experiment box was cleaned with 75% ethanol to remove all odors before entering each mouse. The high-definition camera in the experiment box was used to track the behavior, and the real-time data was analyzed by Shanghai Xin soft software (Shanghai XinRuan Information Technology Co., Ltd., Shanghai, China).

### 2.6. Morris Water Maze Experiment 

Before starting the experiment, the mice were placed in the test room two hours in advance to adapt to the environment. Food-grade titanium dioxide (Jianghu, Shanghai, China) was used for background staining. According to the operation guide (Shanghai Xinruan Information Technology Co., Ltd., Shanghai, China), the mice were released into the water with their head pointing to the tank wall opposite to the platform quadrant. Animals that failed to find the hidden platform within 120 s were gently guided towards it and left there for 30 s. Movement-tracking was recorded by SuperMaze+ software (Shanghai XinRuan Information Technology Co., Ltd., Shanghai, China). The trigger for the ending of data collecting was that the mouse found the platform and successfully stayed on the platform for 3 s. After the training, the water on their bodies was wiped off immediately, and they are transferred to a clean cage for rearing. Each mouse was trained once a day. The platform was kept 2.5 cm below the water surface. For the acquisition training from day 1 to day 5, visible markers were placed on the platform. For the probe test on day 6, the visible markers were removed.

### 2.7. Open Field Test

Open field test was performed according to a method described previously [30], and we adjusted the test duration. The mice were placed into the center of a Plexiglas box (Shanghai Xinruan Information Technology Co., Ltd., Shanghai, China) with a white bottom plate and were allowed to move freely for 5 min. The high-definition camera placed above the box was used to record the activity of each mouse, and the real-time data was processed with the TopScan LITE software (Shanghai Xinruan Information Technology Co., Ltd.). Recording started after the mice were placed in the center of the open field for 5 seconds and the recording ended at 5 min of the test. Before each mouse was placed into the experiment box, the box was wiped with 75% ethanol solution to remove the odor. The experimental environment was kept quiet at 25 °C, and the light was controlled at the same brightness (100 Lux).

### 2.8. Immunohistochemistry 

A total of 3 mice at the age of 10-months from each group (WT, Heterozygote, and Homozygote groups) were randomly selected for the immunohistochemistry experiment. Fresh brain tissue was fixed with 4% paraformaldehyde. After the fixation, the brain tissue was embedded in paraffin and sliced into a thickness of 10 μm. Before adding the primary antibody, the tissue was covered evenly with 3% BSA and blocked for 30 min at room temperature. The primary antibody (IBA-1, 1:500, GB12105; GFAP, 1:1000, GB12096; Aβ, 1:400, GB111197; Servicebio, Wuhan, China) diluted with PBS was added dropwise. The slides were placed in a humidified box and incubated overnight at 4 °C. The slides were placed in PBS (pH7.4) and washed 3 times with shaking on a decolorizing shaker, each time for 5 min. After the slides were dried, the secondary antibody (HRP labeled) corresponding to the primary antibody was dropped to cover the tissue and incubated for 50 min at room temperature. AEC chromogen was used, and the tissue was counterstained with hematoxylin-eosin staining, dehydrated, mounted with glycerin gelatin, and air-dried. The immunohistochemical slices were full-scanned using a full-automatic digital slide scanner (Zeiss, AXIO SCAN Z.1, Carl Zeiss Shanghai Co., Ltd., Shanghai, China). Image-Pro Plus 6.0 software (Media Cybernetics, Inc., Rockville, MD, U.S.A) was used to analyze the mean optical density (MOD) of Aβ, GFAP, and IBA1 expression on one side of the hippocampus as in the previously published article [31,32]. The hippocampal dentate gyrus (DG) was selected for the image display of GFAP. The hippocampal CA3 was selected for the image display of Aβ and IBA1.

### 2.9. DESI-MS Imaging 

Sample preparation: A cryostat (Thermo Scientific™ HM525 NX, Waltham, MA, USA) was used to slice the liver and brain from mice of different genotypes, with a thickness of 15 μm. The sliced tissues adhered onto the same microscope slide, and were stored in a refrigerator at −80 °C until further analysis.

Mass spectrometry conditions: The mass spectrometry was detected by a high-resolution mass spectrometer SYNAPT G2-Si, which was equipped with a desorption electrospray ion source (DESI, Waters, Milford, MA, USA). The data was collected in negative ion mode. The spray capillary voltage was 4.5 kV. The sample cone voltage was 50 V. The ion source temperature was 100 °C. The N_2_ pressure was 4.5 bar. The solvent was 95% methanol + 4.5% water + 0.5% ammonia water. The internal standard leucine enkephalin concentration in the solvent was 6 µg/mL, and the flow rate was 3 µL/min. The scanning range was 100–1000 *m*/*z*. The acquisition mode was the resolution mode.

Imaging scanning and analysis conditions: The spatial resolution was 120 × 120 μm. The scanning speed was 240 μm/s. Before the signal intensity was extracted, the signal of the internal standard leucine enkephalin within 10 s was extracted every 15 min for calibration. The top 1000 substances were subjected to imaging analysis and normalized to the total ion current.

Regions of interest (ROI) were selected on every tissue slice, and the exported MVA data were analyzed to obtain the average signal strength of certain compounds in ROI.

### 2.10. Detection of Substances in Serum

Serum BChE activity was detected using a BChE assay kit (Nanjing Jiancheng Bioengineering Institute, Nanjing, China). Levels of Aβ1-42 and Aβ1-40 were examined with ELISA kits (Meimian, Yancheng, China). A multifunctional microplate reader (TriStar2S LB942, Berthold Technologies, Bad Wildbad, Germany) was used for the final data reading, and the experimental operation was carried out in strict accordance with the product manual.

### 2.11. Data Processing

The *m*/*z* of the substances collected from mass spectrometry imaging was queried through public databases https://hmdb.ca/ (accessed on 13 December 2020) and https://www.lipidmaps.org/ (accessed on 14 December 2020). The substances were determined in conjunction with relevant literature reports [33]. Three animals were selected randomly in each group for the immunohistochemistry and mass spectrometry imaging, and 6–9 mice per group were analyzed in other experiments. GraphPad Prism 7 (GraphPad Software, San Diego, CA, USA) was used in this study. Data were expressed as the mean ± SEM. The data were analyzed by one-way ANOVA test with Tukey’s post hoc test (among multiple groups) or unpaired t test (among two groups). When *p* < 0.05, differences between groups were considered statistically significant.

## 3. Results

### 3.1. Both Gene KO and (R)-Bambuterol Administration Significantly Inhibited Serum Enzyme Activity in Mice

**Figure 1:**
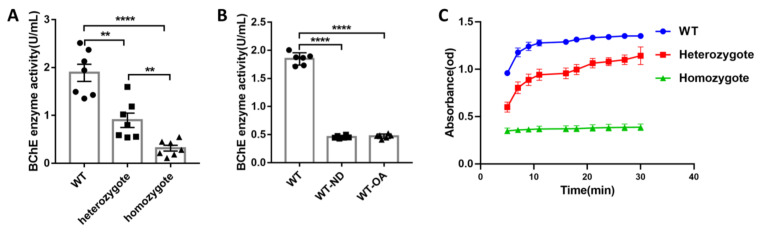
Butyrylcholinesterase (BChE) knockout (KO) and (*R*)-bambuterol administration inhibited the activity of serum BChE enzyme in mice. (**A**) Serum BChE enzyme activity in wild-type (WT) mice and BChE KO mice. (**B**) Serum BChE enzyme activity in WT and oral or nasal administration (R)-bambuterol WT mice. (**C**) Enzymatic reaction rate curve of BChE measured using Ellman test. Data in (**A**,**B**) were shown as mean ± SEM. *n* = 7–9 mice per group. ** *p* < 0.01; **** *p* < 0.0001, one-way ANOVA followed by Tukey’s post hoc test (for **A**,**B**).

To examine the enzymatic activity of BChE in BChE KO mice or inhibition of BChE using (R)-bambuterol, the enzymatic activity of BChE was measured using the serum of 4-month-old mice. The enzymatic activity of BChE in the serum of WT, heterozygote, and homozygote groups was 1.8905 U/mL, 0.9553 U/mL, and 0.3157 U/mL, respectively (Figure 1A). Our data demonstrated that the enzymatic activity of BChE in the heterozygous and homozygous groups was significantly lower than that of the WT (WT vs. heterozgote, *p* = 0.0026; WT vs. homozygote, *p* < 0.0001). In comparison, the enzymatic activity of BChE in the heterozygous group was higher than in the homozygous group (*p* = 0.0057). Theoretically, the heterozygous (BChE^−/−^) mouse should exhibit no BChE activity in the serum; however, low residual butyrylthiocholine hydrolase activity in the test attributed to carboxylesterase ES-10 [34] and contribute to the detection of BChE in the serum of heterozygous mice. In this study, the enzyme activity of BChE in WT, WT-ND, and WT-OA groups was 1.8483, 0.4575, and 0.4713 U/mL, respectively (Figure 1B). In this study, mice with orally or intranasal administration of (*R*)-bambuterol inhibited serum BChE enzyme activity significantly compared to WT (WT vs. WT-ND, *p* < 0.0001; WT vs. WT-OA, *p* < 0.0001;). The enzymatic activity of BChE is no significant difference in WT-ND and WT-OA groups, given that (R)-bambuterol is a strong BChE inhibitor. Our data suggested that (*R*)-bambuterol administered nasally could enter the blood circulation as the (*R*)-bambuterol administered orally did. The enzymatic reaction rate curve of BChe was plotted using the serum of WT, heterozygote, and homozygote groups (Figure 1C). The WT group demonstrated a faster reaction rate than the heterozygous group, and the homozygous group showed the slowest reaction rate. This data supports BChE KO and oral or nasal administration of (*R*)-bambuterol as inhibition of BChE.

### 3.2. Inhibition of BChE Enhanced the Fear Memory, Inhibited the Re-Arousal of Fear, and Accelerated the Fear Extinction in Mice

Freezing is a natural defensive response displayed by rodents when they are afraid. Pairings of sound, light signal (neutral conditioned stimulus), and electric foot-shock (aversive unconditioned stimulus) evoke a fear response in animals during the fear conditioning training. After the fear conditioning training, fear extinction training was carried out in which the animals only receive a conditioned stimulus. The fear conditioning training or fear extinction training can be performed immediately or a few days after the first fear conditioning training. 

**Figure 2:**
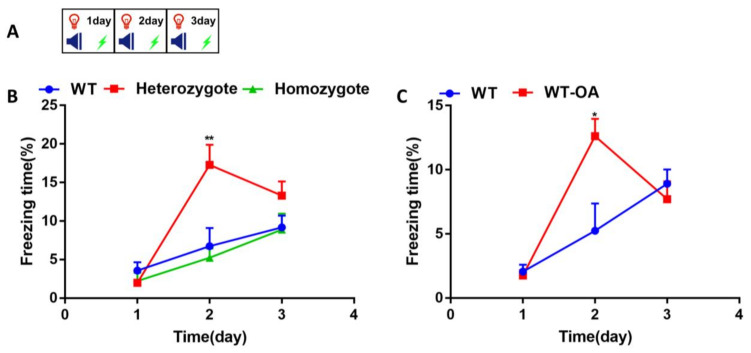
Enhanced fear conditioning memory in BChE heterozygous mice and mice administered (*R*)-bambuterol orally of 4-month-old. (**A**) Experimental design for the fear conditioning training. The freezing time was measured during 180 s of adaptation and 2 cycles of CS + US. (**B**) The percentage of freezing time of wild-type (WT), BChE heterozygous, and BChE KO mice. (**C**) The percentage of freezing time of (*R*)-bambuterol administered through oral (WT-OA). *n* = 7–8 mice per group. Data were represented as mean ± SEM. * *p* < 0.05; ** *p* < 0.01; vs. WT group, one-way ANOVA followed by Tukey’s post hoc test (for **B**) and unpaired t test (for **C**).

To investigate BChE inhibition on fear memory acquisition, fear conditioning training was performed (Figure 2A). The percentage of the freezing time in the 4-month-old WT, heterozygous and homozygous mice was calculated (Figure 2B). On day 1, the freezing time in each group were similar. On day 2, the freezing time in the heterozygous was significantly higher than that of the other two groups (WT vs. heterozgote, *p* = 0.0095; heterozgote vs. homozygote, *p* = 0.0117). On day 3, the freezing time in heterozygous mice decreased, and the freezing time in the other groups were no change. The freezing time of 4-month-old WT mice and WT with orally administration of (*R*)-bambuterol were compared (Figure 2C). On day 1, the freezing time in each group were no change. On day 2, the freezing time in the orally administration group (WT-OA) was significantly higher than that of the control group (WT) (*p* = 0.0108). On day 3, there were no significant differences in freezing time between each group. These results suggest that partial inhibition of BChE enhanced the fear memory formation on day 2 during the continued conditioning training.

**Figure 3:**
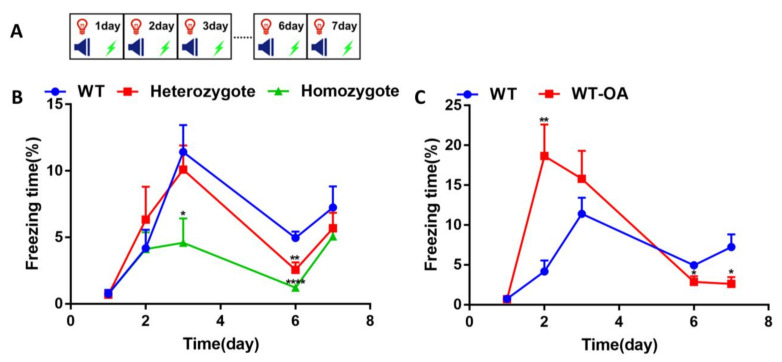
Loss of BChE weakened the re-arousal of fear conditioning memory of 10-month-old mice. (**A**) Experimental design for the re-arousal of fear conditioning training. The freezing time was measured during 180s of adaptation and 2 cycles of CS+US. (**B**) The percentage of freezing time of wild-type (WT), BChE heterozygous, and BChE KO mice. (**C**) The percentage of freezing time of (*R*)-bambuterol administered orally (WT-OA). *n* = 7–8 mice per group. Data were represented as mean ± SEM. * *p* < 0.05; ** *p* < 0.01; **** *p* < 0.0001; vs. WT group, one-way ANOVA followed by Tukey’s post hoc test (for **B**) and unpaired t test (for **C**).

Next, mice were re-exposed to fear conditioning training after several days without disturbance to explore whether BChE inhibition could increase the freezing level. The fear conditioning training was carried out using the 10-month-old mice (Figure 3A). The freezing time in the WT, heterozygous, and homozygous of 10-month-old was similar on the first day (Figure 3B). On the second day, the freezing time in the heterozygote group was slightly longer than that of the other two groups, however no significant difference. On days 3 and 6, the freezing time in homozygous mice was significantly shorter than WT (day 3, WT vs. homozygote, *p* = 0.0447; day 6, WT vs. homozygote, *p* < 0.0001; WT vs. heterozygote, *p* = 0.0033). Using 10-month-old WT and WT mice orally administered (*R*)-bambuterol, the proportion of freezing time of WT was similar to WT mice orally administered (*R*)-bambuterol on the first day (Figure 3C). On the second day, the proportion of freezing time in the orally administered group was significantly higher than that of the control group (*p* = 0.0019). On day 3, there was no significant difference between these groups (*p* = 0.2818). On days 6 and 7, the freezing level in the orally administered group was significantly lower than that of the control group (day 6, *p* = 0.0230; day 7, *p* = 0.0255). These results suggested that inhibition of BChE attenuated the re-arousal of fear conditioning.

**Figure 4:**
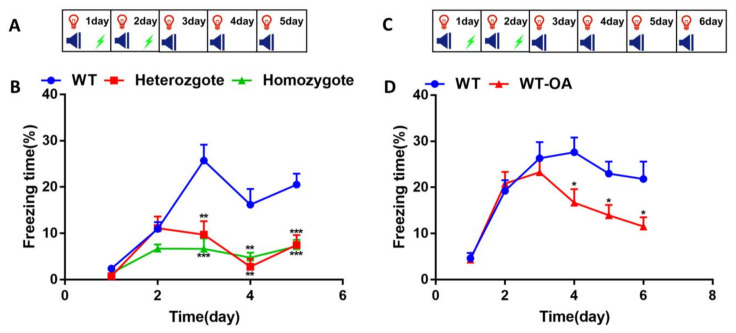
Inhibition of BChE accelerated fear extinction memory in 6 and 8-month-old mice. On days 1–2, the freezing time was measured during 180 s of adaptation and 2 cycles of CS+US; on the other days, the freezing time was measured during 180s of adaptation and 2 cycles of CS. (**A**) Experimental design for the fear extinction training in 6-month-old BChE KO mice. (**B**) The percentage of freezing time in BChE KO mice. (**C**) Experimental design for the fear conditioning memory in (*R*)-bambuterol treated 8-month-old mice. (**D**) The percentage of freezing time in mice with oral administration of (R)-bambuterol (WT-OA). *n* = 7–8 mice per group. Data were shown as mean ± SEM. * *p* < 0.05; ** *p* < 0.01; *** *p* < 0.001; versus WT group, one-way ANOVA followed by Tukey’s post hoc test (for **B**) and unpaired t-test (for **D**).

We then asked whether BChE inhibition could alter the fear extinction in mice. Fear conditioning (on days 1–2) and fear extinction training (on days 3–5) were carried out using WT, heterozygous and homozygous mice at 6-month-old (Figure 4A). On days 1–2, the freezing time of the mice in each group showed no significant differences (Figure 4B). The freezing time on days 3–5 in the homozygous mice was significantly lower than that of the WT mice ((day 3, WT vs. heterozygote, *p* = 0.0022; WT vs. homozygote, *p* = 0.0003); (day 4, WT vs. heterozygote, *p* = 0.0075; WT vs. homozygote, *p* = 0.0081); (day 5, WT vs. heterozygote, *p* = 0.0004; WT vs. homozygote, *p* = 0.0003)). 

To investigate if WT mice orally administered (R)-bambuterol could affect fear extinction, fear conditioning (days 1–2) followed by fear extinction training (days 3–6) was carried out orally and intranasally administered (*R*)-bambuterol aged 8-month-old (Figure 4C). The data of mice intranasally administered (*R*)-bambuterol was showed in Figure S1. The freezing time on days 1–2 were no significant differences in each group; on days 3–6, the freezing time in the oral group was significantly lower than that of the control group (Figure 4D, (day 3, WT vs. WT-OA, *p* = 0.7483; day 4, WT vs. WT-OA, *p* = 0.0354; day 5, WT vs. WT-OA, *p* = 0.0234; day 6, WT vs. WT-OA, *p* = 0.0434)), (Figure S1, (day 3, WT vs. WT-ND, *p* = 0.0261; day 4, WT vs. WT-ND, *p* = 0.0273; day 5, WT vs. WT-ND, *p* = 0.9098; day 6, WT vs. WT-ND, *p* = 0.0098)).

This study investigated the fear conditioning test using 4- and 10-months old mice and the fear extinction test using 6- and 8-months old mice. Differences were observed in the experiments, as mentioned earlier. To investigate if this phenomenon occurs as early as in 4-month-old mice, the combination of fear conditioning, re-exposure, and extinction training was carried out using 4-month-old mice. 

**Figure 5:**
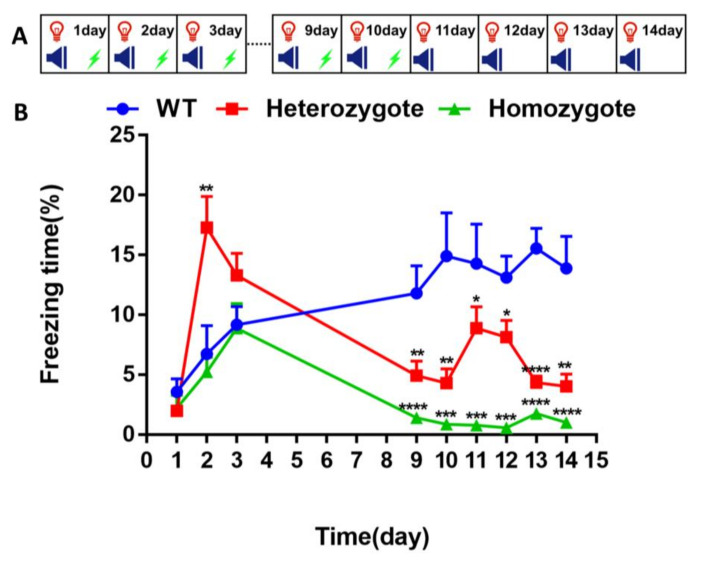
Inhibition of BChE enhanced fear conditioning memory and fear extinction memory in 4-month-old mice. (**A**) Experimental design for the combination of fear conditioning, re-exposure and extinction training. On days 1–3 and 9–10, the freezing time was measured during 180s of adaptation and 2 cycles of CS+US. On days 11–14, the freezing time was measured during 180s of adaptation and 2 cycles of CS. (**B**) The percentage of freezing time of wild-type (WT), BChE heterozygous, and BChE KO mice. *n* = 7–9 mice per group. Data were shown as mean ± SEM. * *p* < 0.05; ** *p* < 0.01; *** *p* < 0.001; **** *p* < 0.0001; versus WT group, one-way ANOVA followed by Tukey’s post hoc test.

The fear conditioning training was on days 1–3 and days 9–10, followed by the fear extinction training on days 11–14 using 4-month-old WT, heterozygous and homozygous mice (Figure 5A). The freezing time in each group was similar on the first day. The freezing time in the heterozygous group was significantly longer than that of the other two groups on the second day. However, there was no significant difference between groups on the third day, and the freezing time in both the heterozygotes and homozygotes were significantly shorter than that of the WT mice on days 9–14 (Figure 5B, (day 9, WT vs. heterozygote, *p* = 0.0062; WT vs. homozygote, *p* < 0.0001); (day 10, WT vs. heterozygote, *p* = 0.0037; WT vs. homozygote, *p* = 0.0002); (day 11, WT vs. heterozygote, *p* = 0.0172; WT vs. homozygote, *p* = 0.0003); (day 12, WT vs. heterozygote, *p* = 0.0252; WT vs. homozygote, *p* < 0.0001); (day 13, WT vs. heterozygote, *p* < 0.0001; WT vs. homozygote, *p* < 0.0001); (day 14, WT vs. heterozygote, *p* = 0.0015; WT vs. homozygote, *p* < 0.0001)].

### 3.3. Inhibition of BChE Resisted Age-Related Spatial Memory Decline in Mice in Morris Water Maze Test

The Morris water maze is mainly used to examine the learning and memory ability of spatial positioning. During the acquisition training, the higher the acquisition slope, the better the spatial memory acquisition ability [35]. 

**Figure 6:**
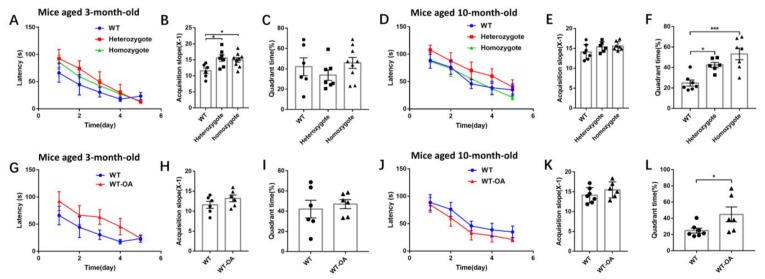
BChE KO and (R)-bambuterol administered mice enhanced the age-related spatial memory using a water maze test. (**A**–**C**) The latency before the successful access to the platform on days 1–5 (the acquisition training), the slope of the latency from day 1 to 5, and the platform quadrant time on day 6 (the probe test) of 3-month-old BChE KO mice. (**D**–**F**) The latency before successful access to the platform on days 1–5, the slope of the latency from day 1 to 5, and the platform quadrant time on day 6 of 10-month-old BChE KO mice. (**G**–**I**) The latency before successful access to the platform on days 1–5, the slope of the latency from day 1 to 5, and the platform quadrant time on day 6 of 3-month-old (*R*)-bambuterol administered mice. (**J**–**L**) The latency before successful access to the platform on days 1–5, the slope of the latency from day 1 to 5, and the platform quadrant time on day 6 of 10-month-old (*R*)-bambuterol administered mice. Data were shown as mean ± SEM. *n* = 6–9 mice per group. * *p* < 0.05; *** *p* < 0.001, one-way ANOVA followed by Tukey’s post hoc test (for **B**,**C**,**E**,**F**) and unpaired t test (for **H**,**I**,**K**,**L**).

To examine spatial positioning’s learning and memory ability, BChE KO mice at 3- and 10-month-old were used. The latency before successful access to the platform during the acquisition training (days 1–5), as shown in Figure 6A. Our findings demonstrated that the acquisition slope of heterozygous and homozygous mice was significantly higher than that of WT mice (Figure 6B) (WT vs. heterozygote, *p* = 0.0319; WT vs. homozygote, *p* = 0.0497). During the probe test, a longer platform quadrant time means the better the memory function. The platform quadrant time on day 6 in 3-month-old BChE KO mice was shown in Figure 6C, and there was no difference in quadrant time between WT, heterozygous and homozygous mice. 

When the mice at 10-month-old, the acquisition slope in heterozygous and homozygous mice was slightly higher than that of the wild-type mice, but no significant difference (Figure 6D,E). The quadrant time of heterozygous and homozygous mice was significantly higher than WT mice, suggesting that the spatial memory function in 10-month-old heterozygous and homozygous mice was better than that of the WT mice (Figure 6F, WT vs. heterozygote, *p* = 0.0209; WT vs. homozygote, *p* = 0.0003). In this study, the quadrant time was decreased from 42.05% in 3-month-old WT mice to 24.78% in 10-month-old WT mice, and BChE KO rescued the age-related decline of spatial memory.

The Morris water maze test was also carried out using the 3-month-old and 10-month-old WT mice administered (*R*)-bambuterol at a dose of 3 mg/kg for two weeks. In 3-month-old mice, the acquisition slope in (*R*)-bambuterol treated mice was slightly higher than the untreated mice (Figure 6G,H for oral treatment and Figure S2A,B for intranasal treatment). There was no difference in quadrant time between each group (Figure 6I and Figure S2C). When the mice were at 10 months of age, the acquisition slope was slightly higher (Figure 6J,K), and the quadrant time were significantly higher (Figure 6L, *p* = 0.0481) in (*R*)-bambuterol administered mice than the WT mice. This study demonstrated that (*R*)-bambuterol treatment rescued the age-related decline of spatial memory. Taken together, both the BChE KO and (*R*)-bambuterol treatment likely reversed the age-related spatial memory decline.

### 3.4. Inhibition of BChE Rescued the Age-Related General Activity Decline in Mice

**Figure 7:**
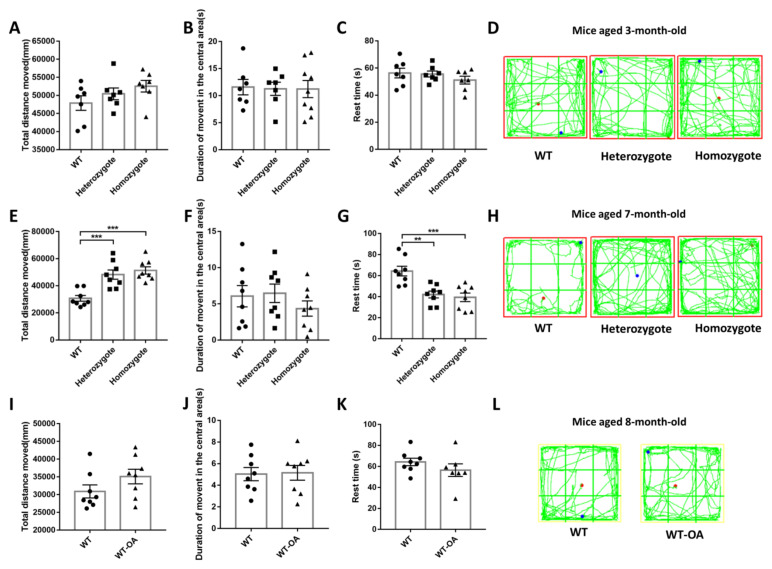
The open field test of 3-month-old, 7-month-old and 8-month-old mice showed that inhibition of BChE enzyme activity rescued the age-related vitality decline. (**A**–**D**) The open field test of 3-month-old WT and BChE KO mice. The total distance, duration of movement in the central area, rest time and representative trajectory diagram were shown. (**E**–**H**) The open field test of 7-month-old WT and BChE KO mice. (**I**–**L**) The open field test of 8-month-old WT mice administered (*R*)-bambuterol through oral route. Data were represented as mean ± SEM. *n* = 7–9 mice per group. ** *p* < 0.01; *** *p* < 0.001, one-way ANOVA followed by Tukey’s post hoc test (for **A**–**C**,**E**–**G**) and unpaired t test (for **I**–**K**).

The open field test is frequently used for the evaluation of general activity levels and anxiety or depression. In the 3-month-old mice, there was no significant difference in general activity between groups (Figure 7A–D). However, in the 7-month-old mice (Figure 7E–H), the total distance moved was decreased in WT mice and BChE KO reversed the decline of general activity ((Figure 7E, WT vs. heterozygote, *p* = 0.0007; WT vs. homozygote, *p* = 0.0001; heterozygote vs. homozygote, *p* = 0.7211); (Figure 7G, WT vs. heterozygote, *p* = 0.0022; WT vs. homozygote, *p* = 0.0007; heterozygote vs. homozygote, *p* = 0.8839)). 

In the 8-month-old WT mice, orally administered of (*R*)-bambuterol reversed the decline of general activity slightly (Figure 7I–L), and intranasally administered (*R*)-bambuterol administration reversed the decline of general activity significantly (Figure S3). The results of the open field test after fear conditioning training also suggest that inhibition of BChE could reverse the age-related general activity decline in mice (Figure S4). Less duration in central is the primary index of anxiety or depression, and the BChE inhibition groups showed the same duration in central compared to the WT group (Figure 7B,F,J), indicating that BChE inhibition did not affect the anxiety level.

### 3.5. Inhibition of BChE Reduced the Optical Density of the Aβ, GFAP and IBA1 in the Hippocampus and Increased the Ratio of Aβ1-42/Aβ1-40 in Serum of 10-Month-Old Mice

**Figure 8:**
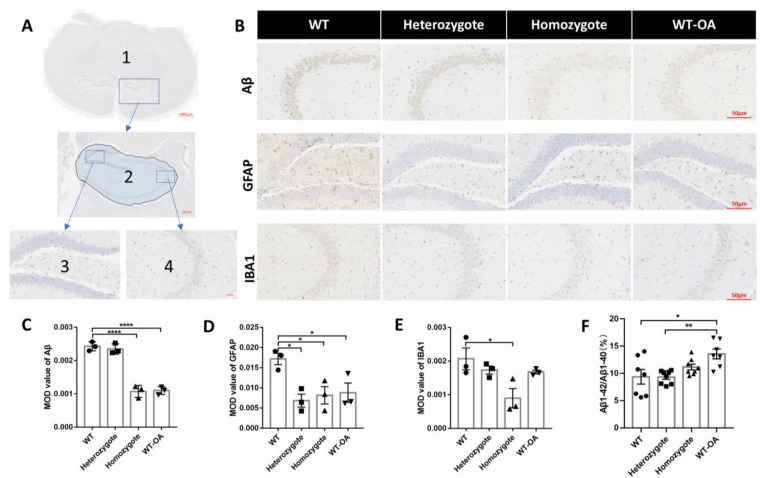
Effect of BChE inhibition on Aβ, GFAP, and IBA1 expression and the serum Aβ1-42/Aβ1-40 in 10-month-old mice. (**A**) The schematic diagram for immunohistochemical analysis: Region 1 was a full-scan display of histochemical slice; region 2 was selected for the average optical density analysis of Aβ, GFAP, and IBA1 expression in the hippocampus; region 3 is the hippocampal DG region and was selected for image display of GFAP, region 4 is the hippocampal CA3 region and was selected for image display of Aβ and IBA1. (**B**) Representative images of the expression of Aβ, GFAP, and IBA1 in the hippocampus of 10-month-old mice. Scale bar: 50 μm. (**C**) Quantitative analysis of the mean optical density of Aβ expression. (**D**) Quantitative analysis of the mean optical density of GFAP expression. (**E**) Quantitative analysis of the mean optical density of IBA1 expression. (**F**) The ratio of Aβ1-42/Aβ1-40 in the serum of 10-month-old mice. N = 3 mice per group, 1 image per animal (for **C**–**E**). *n* = 7–8 mice per group (for **F**). Data were shown as mean ± SEM. * *p* < 0.05; ** *p* < 0.01; **** *p* < 0.0001 vs. WT group, one-way ANOVA followed by Tukey’s post hoc test.

The accumulation of Aβ aggregates in the brain is associated with cognitive impairment. GFAP is a marker of mature astrocytes, and IBA1 is a microglia marker, both of which are closely associated with AD progress. Therefore, we analyzed the Aβ, GFAP, and Iba1 levels in the hippocampus of 10-month-old mice through immunohistochemistry staining. Figure 8A represents the schematic diagram for immunohistochemical analysis. One side of the hippocampus (region 2) was selected for the average optical density analysis of Aβ, GFAP, and IBA1 expression. The hippocampal CA3 contributes to spatial working memory [36], and the increase of GFAP in hippocampal DG is related to stress [37]. Therefore, the DG region (region 3) was selected for image display of GFAP, and the CA3 region (region 4) was selected for image display of Aβ and IBA1 (Figure 8B). Compared with the wild-type mice, the optical density of the Aβ significantly decreases in the hippocampus of homozygous BChE KO mice and (*R*)-bambuterol treated mice (Figure 8C, WT vs. homozygote, *p* < 0.0001; WT vs. WT-OA, *p* = 0.0001). Compared with wild-type mice, the optical density of the GFAP significantly reduces in the hippocampus of BChE KO mice and (*R*)-bambuterol-treated mice (Figure 8D, WT vs. heterozygote, *p* = 0.0134; WT vs. homozygote, *p* = 0.00266; WT vs. WT-OA, *p* = 0.0378). Compared with wild-type mice, the optical density of the IBA1 significantly reduces in the hippocampus of BChE KO mice and (*R*)-bambuterol-treated mice (Figure 8E, WT vs. homozygote, *p* = 0.0161). Meanwhile, the ratios of Aβ1-42/Aβ1-40 in the serum of homozygous BChE KO mice and (*R*)-bambuterol treated mice were higher than that of the control group (Figure 8F, WT vs. homozygote, *p* = 0.4463; WT vs. WT-OA, *p* = 0.0102).

### 3.6. Inhibited BChE Enzyme Activity Increased Glutamine and N-Acetyl-L-Aspartic Acid Content in Mouse Brain

DESI-MS image is a high-throughput and non-target method which can be used to visualize the distribution and richness of endogenous and exogenous substances on the tissue slice by in-situ ionization, without the need for chemical labeling or pretreatment of the tissue [38]. Black represented the background intensity of the mass spectrum signal (Min), and yellow represented the maximum signal in the heatmap. To ensure that the data collection was carried out under the same condition, the tissue samples from different groups were attached to the same glass slide for DESI-MS scanning. The ionization of substances in negative ion mode generally exists in the form of [M−H]^−^ or [M+CI]^−^. 

**Figure 9:**
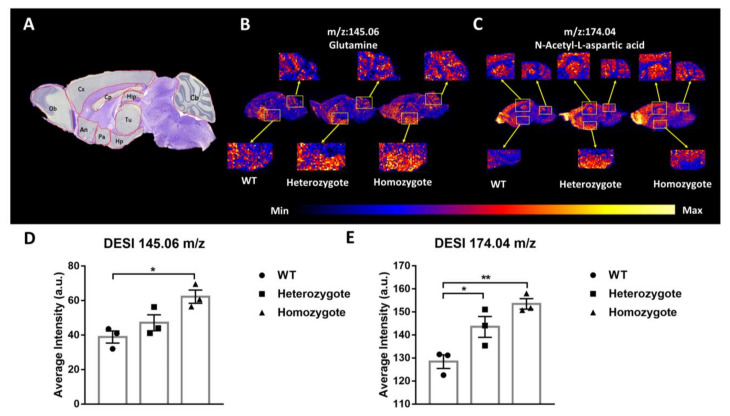
BChE KO increased glutamine and N-acetyl-L-aspartic acid levels in the brain of 10-month-old mice. (**A**) Sagittal section of the brain. Cb, cerebellum; Cx, cerebral cortex; Hip, hippocampus; Cp, corpus callosum; Tu, thalamus; Hp, hypothalamus; Pa, preoptic area; An, nucleus accumbens and Ob, olfactory bulb. (**B**,**C**) Representative images from DESI-MS. The *m*/*z* 145.06 represents L-glutamine, and the *m*/*z* 174.04 represents the N-acetyl-L-aspartic. (**D**) Average intensity (a.u.) of L-glutamine in the Hp and Cb. (**E**) Average intensity (a.u.) of N-acetyl-L-aspartic acid in the Hip, Hp, CX, and Cb. Data were shown as mean ± SEM. *n* = 3 mice per group. * *p* < 0.05; ** *p* < 0.01; vs. WT group, one-way ANOVA followed by Tukey’s post hoc test (for **D**,**E**).

L-glutamine and N-acetyl-L-aspartic acid play important roles in brain metabolism and function [39]. The DESI-MS image showed that the L-glutamine (*m*/*z* = 145.06) levels in the hypothalamus and cerebellum (Cb) of the BChE KO mice were higher than that of the wild-type mice (Figure 9B,D; WT vs. homozygote, *p* = 0.0147; WT vs. heterozgote, *p* = 0.3721). The DESI-MS image also showed that the N-acetyl-L-aspartic acid (*m*/*z* = 174.04) levels in the hippocampus (Hip), hypothalamus [39], cerebral cortex (CX), and cerebellum (Cb) of the BChE KO mice were higher than that of the wild-type mice (Figure 9C,E; WT vs. homozygote, *p* = 0.0047; WT vs. heterozgote, *p* = 0.0454). Our data suggested that BChE enzyme activity inhibition increased glutamine and N-acetyl-L-aspartic acid content in the mouse brain.

## 4. Discussion

This study explores the physiological functions of BChE in the central nervous system and the application prospect of BChE inhibitor (*R*)-bambuterol in the treatment of central nervous system diseases. The serum BChE enzymatic activity in BChE KO mice and BChE inhibitor (*R*)-bambuterol treated mice were first determined. Our results showed that the BChE enzymatic activity of the wild-type group, heterozygous KO group (BChE^+/−^), and homozygous KO group (BChE^−/−^) were decreased gradually. While oral or intranasal administration of (*R*)-bambuterol reduced the BChE activity to the level between heterozygous KO mice and homozygous KO mice (Figure 1). Our study demonstrated that BChE KO mice and the BChE inhibitor (*R*)-bambuterol treated mice could ameliorate the age-related early cognitive decline and accelerate the fear of extinction.

### 4.1. Inhibition of BChE Accelerates Fear Extinction and Enhances the Episodic Memory

The repetition of the conditioned stimulus and unconditioned stimulus several times results in fear memory formation. Repetitive exposure to the conditioned stimulus in the absence of the unconditioned stimulus, a process called fear extinction, induces a progressive reduction of the fear response. Our results indicated that the 4-month-old BChE^+/−^ mice and the 10-month-old BChE^+/−^ mice exhibited increased episodic fear memory on the second day during fear conditioning training (Figure 2). This study was consistent with our group’s previous observation that contextual fear memory was enhanced 24 hours after contextual fear conditioning in the hippocampal CA1 BChE knockdown mice aged 2–3 months [30]. However, as the times of fear conditioning training and fear extinction training was increased, the fear response in BChE^+/−^ and BChE^−/−^ mice were significantly decreased than that of the wild-type mice, suggesting reduced fear re-arousal (Figure 3) and accelerated fear extinction (Figure 4 and Figure 5). Moreover, the fear extinction in BChE^−/−^ mice was faster than BChE^+/−^ mice, indicating a dose-effect relationship between the BChE level and fear extinction (Figure 2, Figure 3, Figure 4 and Figure 5).

The disappearance of fear is the driving mechanism to reduce the fear response, and it is also the basis of exposure-based cognitive behavioral therapy [40]. After experiencing fear memories, they are subsequently strengthened through reintegration or suppressed due to extinction, and this process is considered to be the basis of exposure-based psychotherapy [41]. Involvement of the endocannabinoids in fear extinction makes the endocannabinoid system very attractive for finding effective therapeutics for trauma and stress-related disorders [42]. Enhanced extinction memory retention and reduced return of fear following an overnight fast were negatively correlated with ghrelin plasma levels [43]. BChE inhibition enhanced both fear conditioning memory and fear extinction memory. Therefore, we considered that BChE inhibition could be used to alleviate CNS diseases such as PTSD.

Additionally, depression is one of the complications of posttraumatic stress disorder (PTSD) [44]. A forced swimming test was carried out to evaluate the effect of BChE on depression. When an animal feels it is impossible to escape, it no longer struggles to swim and maintains an immobility state called behavioral despair [45], and antidepressants can significantly shorten the duration of freezing. Our result showed that the immobility time of BChE^+/−^ and BChE^−/−^ mice was significantly lower than that of the wild-type control group (Figure S5), indicating that BChE KO enhanced resistance to despair, BChE inhibition will possibly ameliorate the depression during PTSD treatment.

### 4.2. Inhibition of BChE Ameliorates Risk Factors of Neurodegeneration in 10-Month-Old Mice, and Resists the Age-Related Spatial Memory Decline 

Decline in learning and remembering newly acquired information has been frequently reported in aged animals [13,46], and aging is the leading cause of AD. Our study showed that heterozygous and KO of BChE prevented the age-related decline of spatial memory (Figure 6) and vitality (Figure 7). It is consistent with the finding that the genetic invalidation of BChE augmented spatial learning and memory capacities in mice [35]. Also, Aβ, GFAP, and Iba1 were decreased in the hippocampus of 10-month-old BChE KO mice (Figure 8C–F). GFAP is a marker of mature astrocyte and is increased AD patients’ tissues [47,48]. Iba1 is a marker of mature microglia, and brain IBA1 expression levels are significantly higher in patients with AD than healthy control subjects [49]. 

Moreover, DESI-MSI showed that the N-acetyl aspartic acid (*m*/*z* = 174.04) and L-glutamine (*m*/*z* = 145.06) levels were increased in the brain of 10-month-old BChE KO mice (Figure 9A–E). N-acetyl aspartic acid is synthesized in neuronal mitochondria and transported via axons, its cerebral level correlates with the number of neurons, and is as a putative marker for health, viability, and/or number of neurons [50]. The level of N-acetyl aspartic acid is reduced in the brain of AD patients [51]. L-glutamine is involved in nitrogen transport, regulation of acid-base homeostasis, and catabolic signaling. It is also a substrate for glutathione synthesis, basic building block for proteins, and a potential inhibitory agent for inflammatory cytokine release [52]. An increased of L-glutamine in the brain of BChE KO mice in this study is consistent with our previous publication in which BChE inhibition restored the decreased of L-glutamine in Aβ-42-induced astrocytes [30], and the glutamate–glutamine cycle has a pivotal role in the etiology of AD [53]. Studies also have found that the increase of L-glutamine and N-acetyl aspartic acid can reduce the risk of depression [50,54].

Taken together, the above results showed that the risk factors of neurodegeneration were ameliorated by BChE inhibition. Two mainstream hypotheses about the mechanism of BChE inhibition to treat neurodegenerative diseases. One of the ideas is that BChE inactivates the neurotransmitter ACh. Hence BChE inhibition elevates the level of ACh, which is a strategy in AD therapy. However, our previous study indicates that BChE knockdown in the hippocampal CA1 region exerts no effect on ACh levels, and ChE inhibition affects neurodegenerative disease in an ACh-independent manner when AChE concentrations are no change [30]. 

Another assumption is that BChE affects AD via interacting with Aβ. Aβ is the main pathological feature of AD [55]. Brain sections of demented and nondemented subjects suggested that BChE might transform Aβ from mild to malignant form. BChE KO reduces brain deposition of Aβ in an AD mouse model [56]. The cognitive function of BChE KO mice was not affected by injecting neurotoxic Aβ, while wild-type mice showed impaired cognitive function after injection [7,35,57]. Although the properties of BChE have been reported to be primarily influenced by Aβ. However, inversely, whether BChE promotes or slows the formation of Aβ plaques is still controversial. We observed decreased Aβ level in the hippocampus and increased Aβ-42/Aβ-40 ratio in the blood of 10-month-old BChE KO mice (Figure 8F), indicating the less likely AD occurring [58,59]. Therefore, we support the theory that decreased BChE ameliorates AD by reducing the deposition of Aβ [10]. 

### 4.3. Inhibition of BChE by (R)-Bambuterol Accelerates Fear Extinction and Ameliorates Risk Factors for Age-Related Cognitive Decline

BChE KO mice displayed enhanced memory functions, including fear conditioning memory, fear extinction memory, and spatial memory. To further verify these observations, (*R*)-bambuterol, a potent selective BChE inhibitor, was taken orally or intranasally by wild-type mice. We found that the administration of (*R*)-bambuterol enhanced the fear conditioning memory, accelerated the fear extinction (Figure 3B, Figure 4B and Figure S1B), and reduced the freezing time in the forced swimming test (Figure S5). Besides, administration of (*R*)-bambuterol increased the spatial learning and memory ability in the water-maze test (Figure 6J-L), which were utterly consistent with the results in BChE KO mice, suggesting the potential of bambuterol in the treatment of PSTD, depression and neurodegenerative diseases.

Our results showed that administration methods did not have a decisive influence on (*R*)-bambuterol’s enhancement of fear conditioning, fear extinction, and spatial memory. This discovery implicated that when developing BChE inhibitors for AD treatment, it is unnecessary to require BChE inhibitors to pass through the blood-brain barrier as previously thought.

## 5. Conclusions

This study showed that the BChE inhibition via gene KO and bambuterol administration enhanced the fear conditioning memory, the fear extinction memory and rescued the age-related spatial memory decline and the risk factors of age-related cognitive decline. However, this study is lacking the study of molecular biological mechanisms, which deserves further investigation.

## Figures and Tables

**Table 1:** The primers used for genotyping.

	Sequence (5′–3′)	Template Strand	Length	Start	Stop	Tm	GC%	Self Complementarity	Self 3′ Complementarity
Forward primer	CTGCTCTGCATGCCTTTTGG	Plus	20	62	81	60.11	55.00	6.00	0.00
Reverse primer	ATTTCTGACCCCTGGAAGCC	Minus	20	336	317	59.67	55.00	3.00	1.00
Product primer	275								

**Table 2:** Summary of the mice at different ages for each experiment.

Experiments		BChE Knockout Mice	(*R*)-Bambuterol Treated Mice
		Age	Age
BChE enzyme activity		4 months old	4 months old
Fear conditioning and fear extinction test	Fear conditioning	4 months old	4 months old
	Re-exposure to fear conditioning	10 months old	10 months old
	Fear extinction	6 months old	8 months old
	Combination of fear conditioning, re-exposure and extinction training	4 months old	\
Morris water maze experiment		3 months old	3 months old
		10 months old	10 months old
Open field test		3 months old	\
		7 months old	8 months old
Immunohistochemistry		10 months old	10 months old
DESI-MS imaging		10 months old	\
Detection of substances in serum		10 months old	\

## Data Availability

The data presented in this study are available on reasonable request from the corresponding author.

## Supplementary Materials

The following are available online at https://www.mdpi.com/article/10.3390/biology10050404/s1, Figure S1: Inhibition of BChE accelerated fear extinction memory in 8-month-old mice, Figure S2: Water maze test of 3-month-old (*R*)-bambuterol intranasal administered mice, Figure S3: Inhibition of BChE enzyme activity rescued the age-related vitality decline in mice, Figure S4: The results of the open field experiment after fear conditioning, Figure S5: Inhibition of BChE enzyme activity shortened the immobility time of mice in forced swimming experiments.

## References

[1] Silman I. (2020). The multiple biological roles of the cholinesterases. Prog. Biophys. Mol. Biol..

[2] Santarpia L., Grandone I., Contaldo F., Pasanisi F. (2013). Butyrylcholinesterase as a prognostic marker: A review of the literature. J. Cachexia Sarcopenia Muscle.

[3] Primo-Parmo S.L., Bartels C.F., Wiersema B., van der Spek A.F., Innis J.W., La Du B.N. (1996). Characterization of 12 silent alleles of the human butyrylcholinesterase (BCHE) gene. Am. J. Hum. Genet..

[4] Lockridge O. (2015). Review of human butyrylcholinesterase structure, function, genetic variants, history of use in the clinic, and potential therapeutic uses. Pharmacol. Ther..

[5] Tyurenkov I.N., Velikorodnaya Y.I. (2020). Debatable Aspects of the Role of Butyrylcholinesterase in the Pathogenesisof Neurodegenerative Diseases. Uspehi Sovremennoj Biologii.

[6] Carmona G.N., Schindler C.W., Greig N.H., Holloway H.W., Jufer R.A., Cone E.J., Gorelick D.A. (2005). Intravenous butyrylcholinesterase administration and plasma and brain levels of cocaine and metabolites in rats. Eur. J. Pharmacol..

[7] Duysen E.G., Li B., Lockridge O. (2009). The butyrylcholinesterase knockout mouse a research tool in the study of drug sensitivity, bio-distribution, obesity and Alzheimer’s disease. Expert Opin. Drug. Metab. Toxicol..

[8] Chen V.P., Gao Y., Geng L., Stout M.B., Jensen M.D., Brimijoin S. (2016). Butyrylcholinesterase Deficiency Promotes Adipose Tissue Growth and Hepatic Lipid Accumulation in Male Mice on High-Fat Diet. Endocrinology.

[9] Kurnutala L.N., Rugnath N. (2020). Pseudocholinesterase Deficiency—Is Succinylcholine Still Needed to Facilitate Endotracheal Intubation?. Cureus.

[10] Greig N.H., Utsuki T., Ingram D.K., Wang Y., Pepeu G., Scali C., Yu Q.-S., Mamczarz J., Holloway H.W., Giordano T. (2005). Selective butyrylcholinesterase inhibition elevates brain acetylcholine, augments learning and lowers Alzheimer β-amyloid peptide in rodent. Proc. Natl. Acad. Sci. USA.

[11] Nervo A., Calas A.G., Nachon F., Krejci E. (2019). Respiratory failure triggered by cholinesterase inhibitors may involve activation of a reflex sensory pathway by acetylcholine spillover. Toxicology.

[12] Farooq M.U., Min J.Y., Goshgarian C., Gorelick P.B. (2017). Pharmacotherapy for Vascular Cognitive Impairment. CNS Drugs.

[13] Hamieh A.M., Camperos E., Hernier A.M., Castagné V. (2021). C57BL/6 mice as a preclinical model to study age-related cognitive deficits: Executive functions impairment and inter-individual differences. Brain Res..

[14] Greig N.H., Lahiri D.K., Sambamurti K. (2002). Butyrylcholinesterase: An important new target in Alzheimer’s disease therapy. Int. Psychogeriatr..

[15] Geula C., Mesulam M.M. (1995). Cholinesterases and the Pathology of Alzheimer Disease. Alzheimer Dis. Associated Disorders.

[16] Ha Z.Y., Mathew S., Yeong K.Y. (2020). Butyrylcholinesterase: A Multifaceted Pharmacological Target and Tool. Curr. Protein Pept. Sci..

[17] Pierre A., Van Schuerbeek A., Allaoui W., Van Laere S., Singewald N., Van Eeckhaut A., Smolders I., De Bundel D. (2020). Effects of ghrelin receptor activation on forebrain dopamine release, conditioned fear and fear extinction in C57BL/6J mice. J. Neurochem。.

[18] Yao J., Yuan Y., Zheng F., Zhan C.-G. (2016). Unexpected Reaction Pathway for butyrylcholinesterase-catalyzed inactivation of “hunger hormone” ghrelin. Sci. Rep..

[19] Bukalo O., Nonaka M., Weinholtz C.A., Mendez A., Taylor W.W., Holmes A. (2021). Effects of optogenetic photoexcitation of infralimbic cortex inputs to the basolateral amygdala on conditioned fear and extinction. Behav. Brain Res..

[20] Barricklow J., Blatnik M. (2013). 2-Arachidonoylglycerol is a substrate for butyrylcholinesterase: A potential mechanism for extracellular endocannabinoid regulation. Arch. Biochem. Biophys..

[21] Ney L.J., Akhurst J., Bruno R., Laing P.A.F., Matthews A., Felmingham K.L. (2021). Dopamine, endocannabinoids and their interaction in fear extinction and negative affect in PTSD. Prog. Neuropsychopharmacol. Biol. Psychiatry.

[22] Davis E.A., Wald H.S., Suarez A.N., Zubcevic J., Liu C.M., Cortella A.M., Kamitakahara A.K., Polson J.W., Arnold M., Grill H.J. (2020). Ghrelin Signaling Affects Feeding Behavior, Metabolism, and Memory through the Vagus Nerve. Curr. Biol..

[23] Wu J., Tian Y.G., Wang S.P., Pistolozzi M., Jin Y., Zhou T., Roy G., Xu L., Tan W. (2017). Design, synthesis and biological evaluation of bambuterol analogues as novel inhibitors of butyrylcholinesterase. Eur. J. Med. Chem..

[24] Bosak A., Gazic I., Vinkovic V., Kovarik Z. (2008). Stereoselective inhibition of human, mouse, and horse cholinesterases by bambuterol enantiomers. Chem. Biol. Interact..

[25] Hu T.T., Zhao H.S., Zhang H., Tan W. (2017). The construction of butyrylcholinesterase knockout mouse model using TALENs technology. Heilongjiang J. Anim. Sci. Veterinar. Med..

[26] Gülçin İ., Tel A.Z., Gören A.C., Taslimi P., Alwasel S.H. (2019). Sage (*Salvia pilifera*): Determination of its polyphenol contents, anticholinergic, antidiabetic and antioxidant activities. J. Food Meas. Charact..

[27] Takagi T., Higashi Y., Asai M., Ishii S. (2020). Introduction of a de novo Creb-binding protein gene mutation in sperm to produce a Rubinstein-Taybi syndrome model using inbred C57BL/6 mice. Brain Res..

[28] Diamantopoulou A., Oitzl M.S., Grauer E. (2012). Fear memory for cue and context: Opposite and time-dependent effects of a physiological dose of corticosterone in male BALB/c and C57BL/6J mice. Brain Res..

[29] Morgan M.A., Schulkin J., LeDoux J.E. (2003). Ventral medial prefrontal cortex and emotional perseveration: the memory for prior extinction training. Behav. Brain Res..

[30] Chen S., Lin Z., Tan K.-L., Chen R., Su W., Zhao H., Tan Q., Tan W. (2020). Enhanced Contextual Fear Memory and Elevated Astroglial Glutamate Synthase Activity in Hippocampal CA1 BChE shRNA Knockdown Mice. Front. Psychiatry.

[31] Yao Z., Zhang Z., Zhang J., Cai X., Zhong Z., Huang Y., Qu S. (2021). Electroacupuncture alleviated the depression-like behavior by regulating FGF2 and astrocytes in the hippocampus of rats with chronic unpredictable mild stress. Brain Res. Bull..

[32] Jiang W.W., Wang Q.H., Liao Y.J., Sun Y., Yang R. (2020). The effect of sevoflurane on the spatial recall ability and expression of apolipoprotein E and beta amyloid in the hippocampus in rats. Cell. Mol. Biol..

[33] Leon M., Ferreira C.R., Eberlin L.S., Jarmusch A.K., Pirro V., Rodrigues A.C.B., Favaron P.O., Miglino M.A., Cooks R.G. (2019). Metabolites and Lipids Associated with Fetal Swine Anatomy via Desorption Electrospray Ionization-Mass Spectrometry Imaging. Sci. Rep..

[34] Li B., Duysen E.G., Carlson M., Lockridge O. (2008). The Butyrylcholinesterase Knockout Mouse as a Model for Human Butyrylcholinesterase Deficiency. J. Pharmacol. Exp. Ther..

[35] Maurice T., Strehaiano M., Simeon N., Bertrand C., Chatonnet A. (2016). Learning performances and vulnerability to amyloid toxicity in the butyrylcholinesterase knockout mouse. Behav. Brain Res..

[36] Song D., Wang D.H., Yang Q.H., Yan T.Y., Wang Z., Yan Y., Zhao J., Xie Z., Liu Y.C., Ke Z.J. (2020). The lateralization of left hippocampal CA3 during the retrieval of spatial working memory. Nat. Commun..

[37] Machado-Santos A.R., Alves N.D., Araujo B., Correia J.S., Patricio P., Mateus-Pinheiro A., Loureiro-Campos E., Bessa J.M., Sousa N., Pinto L. (2021). Astrocytic plasticity at the dorsal dentate gyrus on an animal model of recurrent depression. Neuroscience.

[38] Wiseman J.M., Ifa D.R., Zhu Y., Kissinger C.B., Manicke N.E., Kissinger P.T., Cooks R.G. (2008). Desorption electrospray ionization mass spectrometry: Imaging drugs and metabolites in tissues. PNAS.

[39] Noori T., Dehpour A.R., Sureda A., Sobarzo-Sanchez E., Shirooie S. (2021). Role of natural products for the treatment of Alzheimer’s disease. Eur. J. Pharmacol..

[40] Milad M.R., Quirk G.J. (2012). Fear extinction as a model for translational neuroscience: ten years of progress. Annu. Rev. Psychol..

[41] Craske M.G., Fanselow M., Treanor M., Bystritksy A. (2019). Cholinergic Modulation of Exposure Disrupts Hippocampal Processes and Augments Extinction: Proof-of-Concept Study With Social Anxiety Disorder. Biol. Psychiatry.

[42] Gunduz-Cinar O. (2021). The endocannabinoid system in the amygdala and modulation of fear. Prog. Neuropsychopharmacol. Biol. Psychiatry.

[43] Shi L., Deng J.H., Chen S.J., Que J.Y., Sun Y.K., Wang Z., Guo X.J., Han Y., Zhou Y.X., Zhang X.J. (2018). Fasting enhances extinction retention and prevents the return of fear in humans. Transl. Psychiatr..

[44] Sun W., Zhang F.Y., Wang H., Wang C., Zhou Z.G., Zhou Y. (2020). Ginsenoside Rg1 fails to rescue PTSD-like behaviors in a mice model of single-prolonged stress. Biochem. Biophys. Res. Commun..

[45] Snyder J.S., Soumier A., Brewer M., Pickel J., Cameron H.A. (2011). Adult hippocampal neurogenesis buffers stress responses and depressive behaviour. Nature.

[46] Lacreuse A., Parr L., Chennareddi L., Herndon J.G. (2018). Age-related decline in cognitive flexibility in female chimpanzees. Neurobiol. Aging.

[47] Uddin M.S., Stachowiak A., Al Mamun A., Tzvetkov N.T., Takeda S., Atanasov A.G., Bergantin L.B., Abdel-Daim M.M., Stankiewicz A.M. (2018). Autophagy and Alzheimer’s Disease: From Molecular Mechanisms to Therapeutic Implications. Front. Aging Neurosci..

[48] Kamphuis W., Middeldorp J., Kooijman L., Sluijs J.A., Kooi E.J., Moeton M., Freriks M., Mizee M.R., Hol E.M. (2014). Glial fibrillary acidic protein isoform expression in plaque related astrogliosis in Alzheimer’s disease. Neurobiol. Aging.

[49] Sanfilippo C., Castrogiovanni P., Imbesi R., Di Rosa M. (2020). CHI3L2 Expression Levels Are Correlated with AIF1, PECAM1, and CALB1 in the Brains of Alzheimer’s Disease Patients. J. Mol. Neurosci..

[50] Zavorotnyy M., Zöllner R., Rekate H., Dietsche P., Bopp M., Sommer J., Meller T., Krug A., Nenadić I. (2020). Intermittent theta-burst stimulation moderates interaction between increment of N-Acetyl-Aspartate in anterior cingulate and improvement of unipolar depression. Brain Stimul..

[51] Kwo-On-Yuen P.F., Newmark R.D., Budinger T.F., Kaye J.A., Ball M.J., Jagust W.J. (1994). Brain N-acetyl-L-aspartic acid in Alzheimer’s disease: a proton magnetic resonance spectroscopy study. Brain Res..

[52] Luo L.-L., Li Y.-F., Shan H.-M., Wang L.-P., Yuan F., Ma Y.-Y., Li W.-L., He T.-T., Wang Y.-Y., Qu M.-J. (2019). L-glutamine protects mouse brain from ischemic injury via up-regulating heat shock protein 70. CNS Neurosci. Ther..

[53] Bukke V.N., Archana M., Villani R., Romano A.D., Wawrzyniak A., Balawender K., Orkisz S., Beggiato S., Serviddio G., Cassano T. (2020). The Dual Role of Glutamatergic Neurotransmission in Alzheimer’s Disease: From Pathophysiology to Pharmacotherapy. Int. J. Mol. Sci..

[54] Deters B.J., Saleem M. (2021). The role of glutamine in supporting gut health and neuropsychiatric factors. Food Sci. Hum. Wellness.

[55] Park S.A., Han S.M., Kim C.E. (2020). New fluid biomarkers tracking non-amyloid-beta and non-tau pathology in Alzheimer’s disease. Exp. Mol. Med..

[56] Reid G.A., Darvesh S. (2015). Butyrylcholinesterase-knockout reduces brain deposition of fibrillar β-amyloid in an Alzheimer mouse model. Neuroscience.

[57] Furukawa-Hibi Y., Alkam T., Nitta A., Matsuyama A., Mizoguchi H., Suzuki K., Moussaoui S., Yu Q.-S., Greig N.H., Nagai T. (2011). Butyrylcholinesterase inhibitors ameliorate cognitive dysfunction induced by amyloid-beta peptide in mice. Behav. Brain Res..

[58] Seppala T.T., Herukka S.K., Hanninen T., Tervo S., Hallikainen M., Soininen H., Pirttila T. (2010). Plasma A beta 42 and A beta 40 as markers of cognitive change in follow-up: a prospective, longitudinal, population-based cohort study. J. Neurol. Neurosurg. Psychiatry.

[59] Wang D., Di X., Fu L., Li Y., Han X., Wu H., Cai L., Meng X., Jiang C., Kong W. (2016). Analysis of serum beta-amyloid peptides, alpha 2-macroglobulin, complement factor H, and clusterin levels in APP/PS1 transgenic mice during progression of Alzheimer’s disease. Neuroreport.

