# Supplementary material for: Enhancement of Fear Extinction Memory and Resistance to Age-Related Cognitive Decline in Butyrylcholinesterase Knockout Mice and (R)-Bambuterol Treated Mice
Source: Biology (Basel). 2021 May 5;10(5):404. doi: 10.3390/biology10050404 (PMC8147965; doi:10.3390/biology10050404)
Supplement: Supplementary file 1 [file biology-10-00404-s001.zip › biology-1160371-supplementary.pdf]

Supporting information

Enhancement of fear extinction memory and resistance to neurodegeneration in butyrylcholinesterase knockout mice and (*R*)-bambuterol treated mice

Weiwei Liu[1]*, Yan Cao[2,3]*, Yue Lin[1], Keai Sinn Tan[2,3], Haishan Zhao[1], Haihua Guo[1], Wen Tan[2,4#]

1. Institute of Biomedical and Pharmaceutical Sciences, Guangdong University of Technology, Guangzhou 510006, Guangdong, China;
2. YZ Health-tech Inc., Hengqin District, Zhuhai 519000, Guangdong, China
3. School of Pharmacy, Jinan University, Guangzhou 510632, Guangdong, China;
4. Jeffrey Cheah School of Medicine and Health Sciences, Monash University Malaysia, Bandar Sunway 47500, Selangor Darul Ehsan, Malaysia

* These authors contribute equally to the present study.

# Corresponding author: Wen Tan. E-mail: went@gdut.edu.cn.

1. Supplementary methods

1.1 Forced swimming test

Learn from the methods of published articles[1]. The mouse was placed in a glass beaker (20 cm high and 14 cm diameter) with a water depth of 10 cm, and the water temperature was maintained at 25 ℃. The swimming process was recorded with a camera for 6 minutes from the time mice entered the water, and the cumulative immobility time of the mice in the last 4 minutes were calculated.

2. Supplementary figures and legends

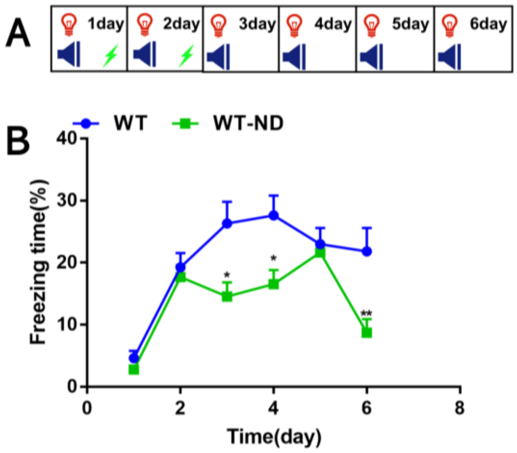

Figure S1. Inhibition of BChE accelerated fear extinction memory in 8-month-old mice. (A) Experimental design for the fear extinction training. (B) The percentage of freezing time of intranasal administration of (*R*)-bambuterol (WT-ND). N = 7-8 mice per group. Freezing time was used as the measure of feared emotional responding. Data were shown as mean ± SEM. *P < 0.05; **P < 0.01; versus WT group, unpaired t test (for B).

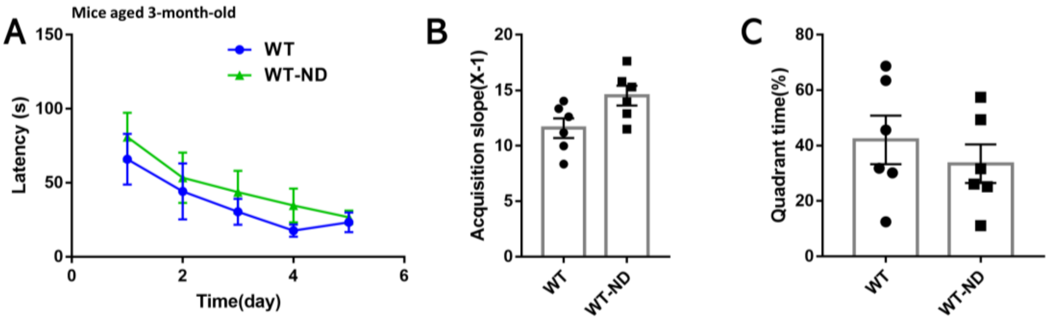

Figure S2. Water maze test of 3-month-old (R)-bambuterol intranasal administered mice. (A-C) The latency before successful access to the platform on days 1-5, the slope of the latency from day 1 to 5, and the platform quadrant time on day 6 of 3-month-old (*R*)-bambuterol administered mice. Data were shown as mean ± SEM. N = 6 mice, unpaired t test (for A-C).

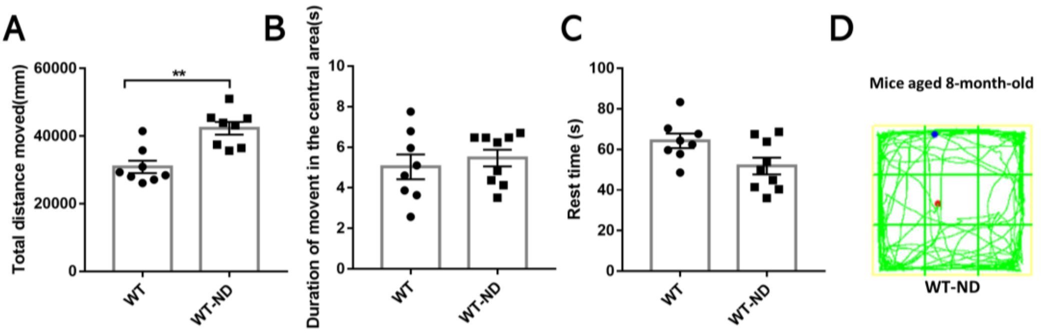

Figure S3. Inhibition of BChE enzyme activity rescued the age-related vitality decline in mice. (A-D) The open field test of 8-month-old WT mice administered (*R*)-bambuterol through intranasal route. Data were represented as mean ± SEM. N = 8 mice. **P < 0.01, unpaired t test (for A-C).

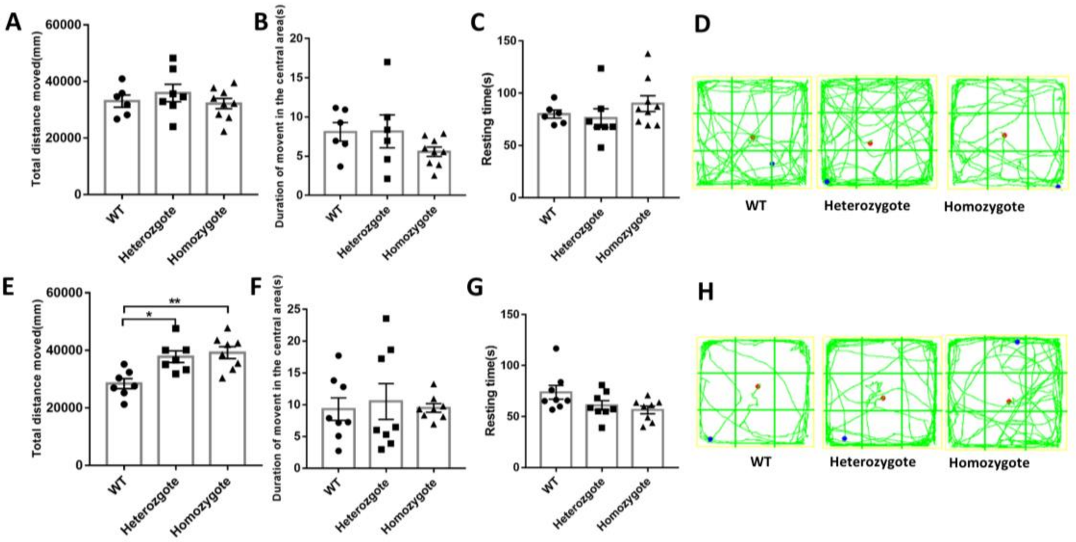

Figure S4. The results of the open field experiment after fear conditioning. (A-D) The open field tests in 4-month-old wild type and BChE KO mice after the fear conditioning. The total distance, duration in the central, rest time and representative trajectory diagram were shown. (E-H) The open field tests in 6-month-old wild type and BChE KO mice after the fear conditioning. The total distance, duration in the central, rest time and representative trajectory diagram were shown. Data were shown as mean ±SEM. N = 6-9 mice per group. *P < 0.05; **P < 0.01, one-way ANOVA followed by Tukey's post-hoc test (for A-C and E-G).

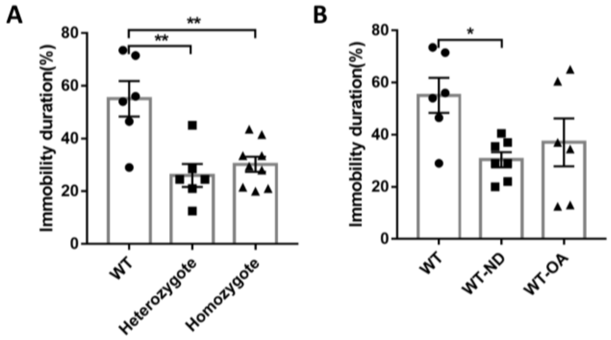

Figure S5. Inhibition of BChE enzyme activity shortened the immobility time of mice in forced swimming experiments. (A) In the 3-month-old, compared with the control group, the immobility time of heterozygous and homozygous mice were significantly shorter. (B)Compared with the control group, intranasal and oral administration of (*R*)-bambuterol reduced the proportion of immobility time in mice. N = 6-7 mice per group. Data were shown as mean ± SEM. *P < 0.05; **P < 0.01. one-way ANOVA followed by Tukey's post-hoc test (for A-B).

1. Cui, L., et al., *Disrupted-in-schizophrenia1 (DISC1) L100P mutation alters synaptic transmission and plasticity in the hippocampus and causes recognition memory deficits.* Molecular Brain, 2016. **9**(1): p. 89.
